# Association of apolipoprotein A1 levels with lumbar bone mineral density and β-CTX in osteoporotic fracture individuals: a cross-sectional investigation

**DOI:** 10.3389/fmed.2024.1415739

**Published:** 2024-07-31

**Authors:** Li-long Feng, Ke Lu, Chong Li, Min-zhe Xu, Yao-wei Ye, Yi Yin, Hui-qiang Shan

**Affiliations:** Department of Orthopedics, Affiliated Kunshan Hospital of Jiangsu University, Suzhou, Jiangsu, China

**Keywords:** osteoporosis, fracture, apolipoprotein A1 (APOA1), bone mineral density (BMD), C-terminal telopeptide of type I collagen (β-CTX), high-density lipoprotein (HDL) osteoporosis, high-density lipoprotein (HDL)

## Abstract

**Background:**

The relationship between the levels of high-density lipoprotein (HDL) and bone mineral density (BMD) is controversial. Furthermore, the specific role of apolipoprotein A1 (APOA1), a primary HDL component, in regulating BMD remains unclear. This study aimed to elucidate the correlation between APOA1 levels and lumbar BMD in patients with osteoporotic fracture (OPF) for novel insights into potential therapeutic strategies against osteoporosis.

**Methods:**

This study included 587 OPF patients enrolled at the Kunshan Hospital, Affiliated with Jiangsu University between January 2017 and July 2022. The patient’s serum APOA1 levels were determined, followed by the assessment of lumbar BMD and C-terminal telopeptide of type I collagen (β-CTX) as outcome variables. The association of APOA1 levels with lumbar BMD and β-CTX was assessed *via* Generalized Estimating Equations (GEE) and spline smoothing plot analyses. A generalized additive model (GAM) helped ascertain non-linear correlations. Moreover, a subgroup analysis was also conducted to validate the result’s stability.

**Results:**

It was observed that APOA1 levels were positively correlated with lumbar BMD (β = 0.07, 95% CI: 0.02 to 0.11, *p* = 0.0045), indicating that increased APOA1 levels were linked with enhanced lumbar BMD. Furthermore, APOA1 levels were negatively related to β-CTX (β = −0.19, 95% CI: −0.29 to −0.09, *p* = 0.0003), suggesting APOA1 might reduce osteolysis. In addition, these findings were robustly supported by subgroup and threshold effect analyses.

**Conclusion:**

This study indicated that increased APOA1 levels were correlated with enhanced lumbar BMD and decreased osteolysis in OPF patients. Therefore, APOA1 may inhibit osteoclast activity to prevent further deterioration in osteoporotic patients. However, further research I warranted to validate these conclusions and elucidate the underlying physiologies.

## Introduction

Osteoporosis (OP) is a prevalent skeletal condition affecting nearly 200 million individuals worldwide (1). It is characterized by decreased bone mineral density (BMD) and increased bone structure degradation, thereby elevating the risk of fracture (2–4). Almost 20% to 30% of osteoporotic patients die within a year of suffering from osteoporotic fractures (OPFs) (5). Furthermore, OP is asymptomatic before an OPF occurs (6). Therefore, early diagnosis and a suitable treatment plan are critical for preventing fractures and re-fractures in OP and OPF patients, respectively.

Previous studies have indicated a correlation between serum HDL levels and bone mass (7). Apolipoprotein A1 (APOA1) is the primary (60–70% of all) apolipoproteins carried by high-density lipoprotein (HDL) (8). It has been observed that APOA1 has anti-atherosclerotic effects (9). Furthermore, recent studies have highlighted that APOA1 regulates bone metabolism homeostasis (10). Moreover, in diabetic individuals, it has been autonomously associated with OP and lumbar BMD but not with HDL (11).

Currently, the relationship between HDL and BMD remains controversial (12) as human epidemiological studies have revealed conflicting results. Some studies have indicated that higher HDL levels can improve bone quality and decrease the risk of OP. However, various studies have suggested an inverse relationship between HDL levels and bone mass (12–14). Therefore, this study aimed to clarify this relationship and determine the precise role of APOA1, a major component of HDL. This study also investigated alterations in the C-terminal telopeptide of type I collagen (β-CTX) in OP patients to assess the bone metabolism pathways and provide new insights into the treatment of OPFs and OP.

## Materials and methods

### Study subjects

**FIGURE 1:**
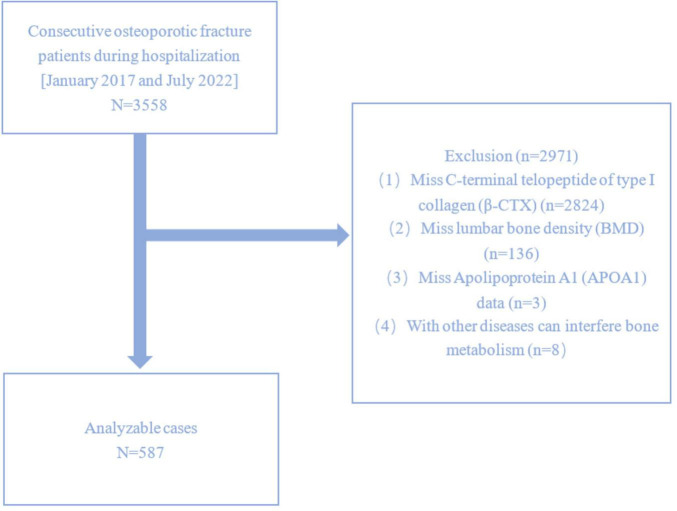
Study flowchart.

This study included 3558 patients diagnosed with OPFs who underwent surgical treatment at Kunshan Hospital Affiliated with Jiangsu University between January 2017 and July 2022. OP is characterized by fragility-related fractures without different metabolic bone disorders despite a normal T-score in BMD. In specific areas such as the femoral neck, lumbar spine, 1/3 (33%) radius, and total hip, OP can be diagnosed without evident fractures with a T score of ≤ −2.5. From 3558 patients, 587 were selected and enrolled (Figure 1). Patients lacking data on β-CTX, lumbar BMD, or APOA1, as well as those who were diagnosed with other diseases that interfere with bone metabolism were excluded. This study acquired ethical clearance from the AKHJU (approval number: 2021-06-015-K01) and adhered to the guidelines of the Helsinki Declaration. The researchers analyzing the data were blinded to the patient-identifiable information. This is an observational study and informed permission as well as anonymized data was acquired from all the patients.

### Measurement of relevant variables

In this observational research, the serum APOA1 levels were assessed as the independent variable using an electro-chemiluminescence immunoassay automated on the LABOSPECT 008AS platform (Hitachi High-Tech Co., Tokyo, Japan). The study outcome variables included lumbar BMD and β-CTX. The lumbar BMD was determined *via* dual-energy X-ray absorptiometry (DXA) using a hologic bone density instrument with dual-energy X-rays (Discovery Wi, Hologic Inc., USA). Furthermore, β-CTX was assessed using an automated electrochemiluminescence immunoassay (ECLIA) (Roche Diagnostics, Mannheim, Germany). The patient’s age and body mass index (BMI; overweight: 24–27.9 kg/m^2^, obese: ≥ 28 kg/m^2^ per the Chinese meta-analysis on obese individuals) (15) were assessed and documented. Alanine aminotransferase (ALT) and uric acid (UA) levels were determined with an enzymatic colorimetric method using the Beckman AU5800 biochemical analyzer pipeline. Moreover, triglyceride levels were assessed via the Guaiacol Peroxidase-Peroxidase Oxidation of Phenol (GPO-POD) method with the biochemical analyzer pipeline, Beckman AU5800. In addition, the Sysmex XN-10 (B4) hematology analyzer pipeline was employed to assess hemoglobin, monocyte, and platelet levels. The individuals’ parathyroid hormone (PTH) levels were determined with the Beckman DXI800 using a chemiluminescent assay. Furthermore, the patient’s HDL and LDL levels were assessed using the direct method with the biochemical analyzer pipeline, Beckman AU5800. Lastly, the automated coagulation analyzer CN-6000 and the Beckman AU5800 biochemical analyzer pipeline were employed to assess fibrinogen and albumin levels, respectively. FAR was calculated by dividing the fibrinogen and albumin levels for each patient. For the above analyses, the patient’s fasting blood samples were collected within 24 hours after admission. A skilled operator collected the samples using identical equipment according to established protocols.

### Covariate analyses

Based on the previous literature (11, 16, 17), clinical guidelines (18), data within our database, and the results of previous clinical work by our team, following variables were selected as covariates: age, BMI, triglycerides, ALT, UA, diabetes, hypertension, monocyte, hemoglobin, platelet, PTH, HDL, LDL (low-density lipoprotein), neutrophil and FAR (fibrinogen/albumin ratio).

### Statistical analyses

The demographic, laboratory, and clinical data are demonstrated as medians (25*^th^* and 75*^th^* percentile) or means with standard deviations (SD). Furthermore, the categorical data are presented as frequencies (percentages). Fisher’s exact or Pearson’s chi-square tests were performed for the univariate analysis of categorical data. Moreover, not-normally and regularly distributed continuous data were analyzed via Independent sample Mann-Whitney U tests and *t*-tests, respectively. In addition, univariate analyses were performed to determine the correlation of OPF patient’s characteristics with lumbar BMD and β-CTX.

The study utilized Generalized Estimating Equations (GEE) to elucidate the independent association between Lipoprotein A levels, lumbar BMD, and β-CTX in OPF patients while controlling for confounders. The models constructed comprised of unadjusted (Model 1), little adjusted (Model 2), and wholly adjusted (Model 3) models. Initially, the variance inflation factor (VIF) was utilized for assessing the covariance co-linearity. Then, these variables were corrected based on: (1) a substantial alteration within the matched odds ratio (OR) of at least 10%, after including or excluding covariates from the base or complete models, and (2) covariates either satisfied criteria 1 or demonstrated a *p*-value of < 0.1 within the univariate model. Models 2 and 3 were built using Criterion 1 and 2 to adjust for covariates. Three models were created, as outlined below: Model 1 was unadjusted; Model 2 (minimally adjusted model) accounted for age and BMI, while Model 3 accounted for age, BMI, triglycerides, ALT, UA, diabetes, hypertension, monocyte, hemoglobin, platelet, and PTH.

The probable non-linear correlations were assessed via a generalized additive model (GAM). A smoothing curve was generated using a two-piecewise linear regression model to determine the threshold effects. When an evident ratio was visible, the inflection point of these curves was determined using a recursive technique based on a model of maximum likelihood (19). The robustness and subgroup variability of the study patients were evaluated post-stratification according to specific variables. The subgroup interactions and modifications were employed for the likelihood ratio test (LRT).

All the statistical evaluations were performed using the R Foundation packages^1^ and EmpowerStats from X&Y Solutions, Inc., MA, USA.^2^ *p* < 0.05 was deemed as the significance level for a two-sided test.

## Results

### Features of patients

The baseline features of OPF patients (*n* = 3558) hospitalized within established APOA1 quartiles between January 2017 and July 2022 have been highlighted in Table 1. There were 31.93% males and 68.07% females with an average age of 69.18 ± 11.24 years, mean lumbar BMD of 0.74 ± 0.15 g/cm^2^, mean β-CTX of 0.53 ± 0.28 ng/mL and mean APOA1 of 1.22 ± 0.24 ng/mL. Patients were stratified into APOA1 quartiles (0.29–1.05, 1.06–1.19, 1.20–1.34, and 1.35–2.49 ng/mL), and interquartile differences within lumbar BMD, β-CTX, age, BMI, triglycerides, ALT, UA, diabetes, hypertension, monocyte, hemoglobin, platelet, PTH, HDL, LDL, neutrophil and FAR. It was observed that, higher APOA1 quartile patients had substantially increased lumbar BMD (Q1: 0.73 ± 0.15 g/cm^2^; Q2: 0.72 ± 0.15 g/cm^2^; Q3: 0.75 ± 0.17 g/cm^2^; Q4: 0.77 ± 0.15 g/cm^2^, *p* = 0.004), while those in elevated APOA1 quartiles had lower β-CTX (Q1: 0.58 ± 0.35 ng/mL; Q2: 0.55 ± 0.28 ng/mL; Q3: 0.53 ± 0.25 ng/mL; Q4: 0.51 ± 0.24 ng/mL).

**TABLE 1:** Patient characteristics based on apolipoprotein A quartiles.

Apolipoprotein A1 quartile	Q1	Q2	Q3	Q4	*P*-value	*P*-value*
N	535	568	542	563		
Lumber BMD, g/cm^2^	0.73 ± 0.15	0.72 ± 0.15	0.75 ± 0.17	0.77 ± 0.15	0.004	0.002
β-CTX, ng/mL	0.58 ± 0.35	0.55 ± 0.28	0.53 ± 0.25	0.51 ± 0.24	0.184	0.564
Age, year	70.17 ± 11.57	68.96 ± 11.22	68.76 ± 10.55	68.45 ± 11.25	0.061	0.053
BMI categorical, *N* (%)	22.82 ± 3.44	23.04 ± 3.43	23.17 ± 3.45	22.87 ± 3.33	0.304	0.432
Triglycerides, mmol/L	1.16 ± 0.79	1.22 ± 0.90	1.29 ± 0.87	1.26 ± 1.19	0.107	0.136
ALT, IU/L	21.59 ± 15.10	23.25 ± 30.04	23.33 ± 19.96	22.05 ± 13.30	0.411	0.066
UA, μmol/L	295.80 ± 95.95	284.30 ± 90.79	275.92 ± 88.16	271.41 ± 87.46	<0.001	<0.001
Monocyte, × 10^9^/L	0.54 ± 0.26	0.52 ± 0.49	0.47 ± 0.24	0.46 ± 0.22	<0.001	<0.001
Hemoglobin, g/L	121.25 ± 20.68	126.69 ± 18.22	127.09 ± 16.94	128.63 ± 14.89	<0.001	<0.001
Platelet, × 10^9^/L	172.35 ± 67.79	178.18 ± 61.20	175.37 ± 61.16	180.52 ± 55.88	0.146	0.010
HDL, mmol/L	1.07 ± 0.19	1.26 ± 0.19	1.40 ± 0.21	1.64 ± 0.30	<0.001	<0.001
LDL, mmol/L	2.26 ± 0.76	2.54 ± 0.75	2.66 ± 0.72	2.72 ± 0.75	<0.001	<0.001
Neutrophil, × 10^9^/L	6.42 ± 3.24	6.29 ± 2.99	6.11 ± 2.83	6.48 ± 3.05	0.194	0.225
FAR	0.08 ± 0.03	0.07 ± 0.02	0.07 ± 0.02	0.07 ± 0.02	<0.001	<0.001
PTH, pg/mL	16.29 ± 12.72	14.81 ± 11.42	14.30 ± 8.84	13.25 ± 7.44	<0.001	0.002
Diabetes					0.153	–
No	511 (95.51%)	555 (97.71%)	521 (96.13%)	537 (95.38%)		
Yes	24 (4.49%)	13 (2.29%)	21 (3.87%)	26 (4.62%)		
Hypertension					0.943	–
No	465 (86.92%)	495 (87.15%)	469 (86.53%)	484 (85.97%)		
Yes	70 (13.08%)	73 (12.85%)	73 (13.47%)	79 (14.03%)		

*Kruskal-Wallis rank test for continuous variables, Fisher exact for categorical variables with expects < 10. APOA1, apolipoprotein A1; Lumber BMD, lumbar spine bone density; β-CTX, C-terminal telopeptide of type I collagen; BMI, body mass index; ALT, alanine aminotransferase; UA, uric acid; PTH, parathyroid hormone; HDL, high-density lipoprotein; LDL, low-density lipoprotein; FAR, fibrinogen/albumin ratio.

### Factors correlated with lumbar BMD and β-CTX based on univariate analysis

The univariate analysis indicated a significant correlation between lumbar BMD, β-CTX, and other factors including age, BMI, triglycerides, ALT, UA, diabetes, hypertension, monocyte, hemoglobin, platelet, and PTH, particularly with APOA1 (Table 2).

**TABLE 2:** Univariate analyses of factors associated with Lumber BMD and β-CTX.

	Statistics	Lumber BMD β (95%CI) *P*-value	β -CTX β (95%CI) *P*-value
APOA1, ng/mL	1.22 ± 0.24	0.07 (0.02, 0.12) 0.0027	−0.14 (−0.24, −0.04) 0.0080
Age, year	69.18 ± 11.24	−0.00 (−0.00, −0.00) < 0.0001	0.00 (−0.00, 0.00) 0.5157
BMI categorical, N (%)	23.00 ± 3.38	0.01 (0.01, 0.01) < 0.0001	0.00 (−0.00, 0.01) 0.6053
Triglycerides, mmol/L	1.23 ± 0.95	0.01 (−0.00, 0.02) 0.1675	−0.01 (−0.03, 0.02) 0.5471
ALT, IU/L	23.62 ± 22.32	−0.00 (−0.00, 0.00) 0.8580	−0.00 (−0.00, −0.00) 0.0040
UA, μmol/L	284.80 ± 92.36	−0.00 (−0.00, 0.00) 0.3764	−0.00 (−0.00, −0.00) 0.0014
Monocyte, × 10^9^/L	0.51 ± 0.30	0.01 (−0.02, 0.04) 0.6119	−0.17 (−0.26, −0.07) 0.0005
Hemoglobin, g/L	125.70 ± 18.45	−0.00 (−0.00, 0.00) 0.6943	−0.00 (−0.00, −0.00) 0.0243
Platelet, × 10^9^/L	177.62 ± 62.32	−0.00 (−0.00, 0.00) 0.4770	0.00 (0.00, 0.00) 0.0021
**Diabetes**			
No	3413 (95.92%)	Reference	Reference
Yes	145 (4.08%)	0.04 (−0.01, 0.08) 0.0882	0.02 (−0.08, 0.13) 0.6342
**Hypertension**			
No	3048 (85.67%)	Reference	Reference
Yes	510 (14.33%)	0.01 (−0.01, 0.04) 0.2196	0.05 (−0.01, 0.11) 0.1063

APOA1, apolipoprotein A1; Lumber BMD, lumbar spine bone density; β-CTX, C-terminal telopeptide of type I collagen; BMI, body mass index; ALT, alanine aminotransferase; UA, uric acid; PTH, parathyroid hormone; HDL, high-density lipoprotein; LDL, low-density lipoprotein; FAR, fibrinogen/albumin ratio.

### The association of APOA1 levels with Lumbar BMD and β-CTX

Three models assessed the link between APOA1, lumbar BMD, and β-CTX in OPF patients (Table 3). The first analysis, Model 1, indicated a significant association of APOA1 with lumbar BMD (β = 0.07, 95% CI = 0.02 to 0.12, *p* = 0.0027), and β-CTX (β = −0.14, 95% CI = −0.24 to −0.04, *p* = 0.0080). After controlling for age and BMI, Model 2 indicated comparable correlations, specifically between APOA1 and lumbar BMD (β = 0.06, 95% CI = 0.02 to 0.11, *p* = 0.0050, and APOA1 and β-CTX (β = −0.14, 95% CI = −0.24 to −0.04, *p* = 0.0072). Model 3 showed comparable correlations between APOA1 and lumbar BMD (β = 0.07, 95% CI = 0.02 to 0.11, *p* = 0.0045), and APOA1 and β-CTX (β = −0.19, 95% CI = −0.29 to −0.09, *p* = 0.0003), after controlling for age, BMI, triglycerides, ALT, UA, diabetes, hypertension, monocyte, hemoglobin, platelet, and PTH.

**TABLE 3:** Association between APOA1 and BMD and β-CTX in different models.

	Model 1^a^N = 718 β (95%CI) *P*-value	Model 1^b^N = 718 β (95%CI) *P*-value	Model 1^c^N = 718 β (95%CI) *P*-value
BMD	0.07 (0.02, 0.12) 0.0027	0.06 (0.02, 0.11) 0.0050	0.07 (0.02, 0.11) 0.0045
β-CTX	−0.14 (−0.24, −0.04) 0.0080	−0.14 (−0.24, −0.04) 0.0072	−0.19 (−0.29, −0.09) 0.0003

^a^No adjustment.

^b^Adjusted for age, BMI.

^c^Adjusted for age, BMI, triglycerides, ALT, UA, monocyte, hemoglobin, platelet, PTH, diabetes, and hypertension. APOA1, apolipoprotein A1; Lumber BMD, lumbar spine bone density; β-CTX, C-terminal telopeptide of type I collagen; BMI, body mass index; ALT, alanine aminotransferase; UA, uric acid; PTH, parathyroid hormone; HDL, high-density lipoprotein; LDL, low-density lipoprotein; FAR, fibrinogen/albumin ratio.

The strength and reliability of Model 3 were validated by additional subgroup analyses, which categorized OPF patients according to age, BMI, triglycerides, ALT, UA, diabetes, hypertension, monocyte, hemoglobin, platelet, and PTH levels. The analyses were adjusted to incorporate the remaining un-stratified covariates. Thus, similar patterns were observed without discernible interactions because of stratification (*p* > 0.05, Supplementary Table 1).

### Spline smoothing graph and threshold exploration

**FIGURE 2:**
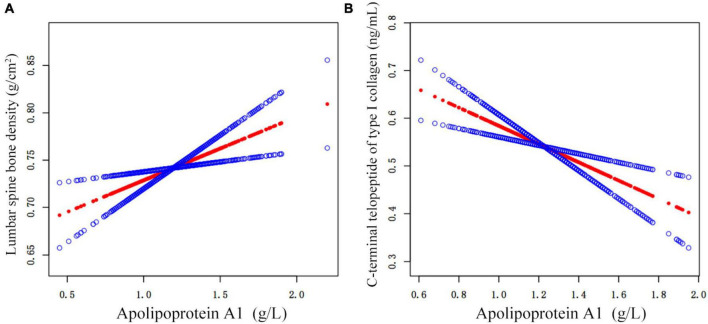
APOA1 levels are connected with lumbar BMD **(A)** β-CTX **(B)** on adjusted smoothed curves. The top and bottom curves demonstrate the span of the 95% confidence interval. In contrast, the center curve highlights the linkage between APOA1 and lumbar BMD and β-CTX. Age, BMI, triglycerides, ALT, UA, monocyte, hemoglobin, platelet, diabetes, and hypertension were adjusted within the model. APOA1, apolipoprotein A1; Lumber BMD, lumbar spine bone density; β-CTX, C-terminal telopeptide of type I collagen; BMI, body mass index; ALT, alanine aminotransferase; UA, uric acid; PTH, parathyroid hormone; HDL, high-density lipoprotein; LDL, low-density lipoprotein; FAR, fibrinogen/albumin ratio.

Different graphs assessed the linearity or non-linearity of the correlation between lumbar BMD-APOA1 and β-CTX-APOA1 for 769 and 587 individuals, respectively (Figure 2). The threshold effect analysis further involved age adjustments, BMI, triglycerides, ALT, UA, diabetes, hypertension, monocyte, hemoglobin, platelet, and PTH (Table 4). However, a direct correlation of APOA1 levels with lumbar BMD and β-CTX could be observed. The final model adjusted for age, BMI, triglycerides, ALT, UA, diabetes, hypertension, monocyte, hemoglobin, platelet, and PTH level counts indicated an impact size of 0.07 (95% CI = 0.02 to 0.12, *p* = 0.0027) and −0.19 (95% CI = −0.29 to −0.09, *p* = 0.0003). In addition, the curve fitting-based threshold effect analysis did not depict a non-linear correlation (Table 4) without proven inflection points (*p* > 0.05).

**TABLE 4:** Threshold analyses examining the relationship between APOA1 levels and BMD, β-CTX.

	Model 3^a^	
	**Lumber BMD β (95% CI) *P*-value**	**β -CTX β (95% CI) *P*-value**
**Model A^b^**		
One line slope	0.07 (0.02, 0.11) 0.0045	−0.19 (−0.29, −0.09) 0.0003
**Model B^c^**		
APOA1 turning point (K), g/L	1.17	0.91
<K	0.01 (−0.09, 0.11) 0.8542	0.32 (−0.64, 1.29) 0.5133
>K	0.10 (0.03, 0.17) 0.0058	−0.21 (−0.32, −0.10) 0.0002
Slope 2–Slope 1	0.09 (−0.05, 0.24) 0.2180	−0.53 (−1.53, 0.46) 0.2948
BMD value at K, g/cm^2^ or β-CTX value at K, ng/mL	0.73 (0.72, 0.75)	0.60 (0.56, 0.64)
LRT test^d^	0.214	0.289

^a^Adjusted for age, BMI, triglycerides, ALT, UA, diabetes, hypertension, monocyte, hemoglobin, platelet and PTH.

^b^Linear analysis, *P*-value < 0.05 indicates a linear relationship.

^c^Non-linear analysis.

^d^*P*-value < 0.05 means Model B is significantly different from Model A, which indicates a non-linear relationship APOA1, apolipoprotein A1; Lumber BMD, lumbar spine bone density; β-CTX, C-terminal telopeptide of type I collagen; BMI, body mass index; ALT, alanine aminotransferase; UA, uric acid; PTH, parathyroid hormone; HDL, high-density lipoprotein; LDL, low-density lipoprotein; FAR, fibrinogen/albumin ratio.

## Discussion

The OP is an increasing public health concern (20–23). A recent cross-sectional research on Chinese individuals indicates an OP prevalence of 5.0% in males and 20.6% in women aged 40 years and above (24). OP significantly increases the financial burden of patients who endure severe bone fractures and physical pain (25). OPFs are common causes of morbidity and death among older adults (26–28), with vertebral fractures being the most common OPF (29). Therefore, in this study, lumbar BMD was selected as the outcome variable. Emerging evidence has depicted that aberrant lipid metabolic pathways can affect osteoblast differentiation, leading to bone mass loss (11). Furthermore, it has been indicated that serum HDL is associated with BMD; however, it remains controversial (12). Several studies have suggested that HDL is inversely associated with BMD (13, 14, 30). However, some studies reported that HDL is positively linked with BMD (13, 31). Recently, APOA1, the main HDL component, has been independently associated with BMD (11).

Therefore, this study used APOA1 as an exposure variable within a Chinese osteoporotic population. APOA1, a 243-amino acid polypeptide (32), is the primary apolipoprotein HDL component and a common protein in human plasma (33). Most of the ApoA1 protein (nearly 80%) is secreted by hepatocytes, while only a small amount (20%) is secreted by the intestinal epithelial cells (34). Similar to HDL, APOA1 could have antioxidant and anti-inflammatory properties (35). It has been observed to play a role in atherosclerotic diseases, including anti-atherosclerotic, anti-inflammatory, anti-apoptotic, and anti-thrombotic effects (36, 37). *In vivo* mice experiments have revealed that APOA1 deficiency affects bone metabolism via multiple mechanisms (10, 38, 39). Moreover, it is a versatile protein that positively influences various diseases (40).

The current study indicated that APOA1 is autonomously linked with lumbar BMD. Furthermore, its levels were positively correlated with lumbar BMD after adjusting for relevant variables (β = 0.07, 95% CI = 0.02 to 0.11, *p* = 0.005). The curve-fitting between APOA1 and lumbar BMD indicated a high linear relationship (Figure 2). These results indicate a significantly reliable arrangement with no evidence of stratification-linked exchanges (*p* > 0.05, Supplementary Table 1). Based on these findings, it was inferred that as APOA1 enhances, lumbar BMD also increases in OPF patients.

Moreover, a bone resorption (β-CTX) indicator was selected as the other outcome variable to assess the mechanisms affecting BMD changes. B -CTX is an indicator of bone metabolism (41–43). Clinical studies have indicated that enhanced β-CTX levels are linked with increased bone resorption, higher risk of fractures, and accelerated bone loss (44). The International Federation of Clinical Chemistry (IFCC) and the International Osteoporosis Foundation (IOF) have suggested that serum β-CTX could become a reference bone turnover marker (BTM) (45).

Therefore, in this study, it was observed that APOA1 levels were significantly correlated with β-CTX and indicated a linear negative relationship (β = −0.19, 95% CI = −0.29 to −0.09, *p* < 0.001). These outcomes delivered a significantly reliable array with no proof of stratification-linked interrelationships (*p* > 0.05, Supplementary Table 1). These findings suggest that β-CTX decreases with enhancing APOA1, highlighting osteolysis decline, and validating the conclusion that lumbar BMD increases with APOA1, indicating a potential mechanism that elevates lumbar BMD.

A recent *in vivo* mice study confirmed that APOA1 deficiency can influence bone metabolism by altering the phenotypic and molecular characteristics of bone marrow adipocytes (38, 39). Furthermore, APOA1 deficiency modified the bone cell precursor population, thereby increasing adipoblast and decreasing osteoblast syntheses (10).

Overall, these data indicated that APOA1 elevates BMD and prevents further deterioration of OP by decreasing osteoclast activity, depicting clinical implications. Therefore, based on the results of this study, clinicians should detect and maintain APOA1 in osteoporotic patients to decrease osteolysis and enhance BMD.

The study screened OPF patients *via* accurate statistical models and adjustment of essential confounding factors. Per our knowledge, this is the first Chinese investigation to investigate APOA1 in OPF patients to evaluate the relationship between APOA1 and lumbar BMD. Furthermore, this study not only provides novel insights into the association between HDL and BMD but also highlights innovative treatment strategies for patients.

However, the current study has limitations. (1) Several studies have identified the connection between HDL and BMD; however, many co-founding variables, such as age, sex, and ethnicity, were not focused. (2) This study only included OPF patients, and whether the current findings apply to the general population requires further exploration. (3) Due to the study’s cross-sectional design, the correlation between APOA1, lumbar BMD, and β-CTX as well as a causal relationship between APOA1 levels and variations in BMD and β-CTX could not be assessed. Without longitudinal data, it is not possible to monitor changes in bone density over time or evaluate the long-term effects of APOA1 levels on bone health. (4) The results suggested that the enhanced lumbar BMD was correlated with APOA1, which requires further investigation. Increased APOA1 reduced β-CTX, indicating that decreased osteolysis is a possible cause. Further studies are warranted to confirm these findings and illustrate the potential mechanisms.

## Conclusion

In summary, the correlation between HDL and BMD has been controversial; however, this study indicated a positive correlation between APOA1 levels, a major HDL component, and lumbar BMD, indicating that APOA1 prevents further deterioration in OPF patients. Moreover, it was observed that APOA1 may produce an anti-OP effect by suppressing osteoclast action. Further investigations are warranted to validate these results and highlight the underlying physiologies.

## Data availability statement

The original contributions presented in this study are included in the article/Supplementary material, further inquiries can be directed to the corresponding author.

## Ethics statement

The studies involving humans were approved by the IRB of Affiliated Kunshan Hospital of Jiangsu University. The studies were conducted in accordance with the local legislation and institutional requirements. The participants provided their written informed consent to participate in this study.

## Author contributions

Ll-F: Writing−original draft, Writing−review and editing. KL: Writing−original draft, Writing−review and editing. CL: Writing−original draft, Writing−review and editing. H-qS: Writing−review and editing. M-zX: Writing−review and editing. Y-wY: Writing−review and editing. YY: Writing−review and editing.

## Footnotes

1
www.R-project.org

2
www.empowerstats.com
