# Supplementary material for: Association of apolipoprotein A1 levels with lumbar bone mineral density and β-CTX in osteoporotic fracture individuals: a cross-sectional investigation
Source: Front Med (Lausanne). 2024 Jul 31;11:1415739. doi: 10.3389/fmed.2024.1415739 (PMC11322117; doi:10.3389/fmed.2024.1415739)
Supplement: Supplementary file 1 [file Table_1.docx]

**Table S1.** Subgroup analyses exploring the association between APOA1 levels and lumbar BMD and β-CTX

| Subgroup | N | lumbar BMD  β (95%CI) *P-*value | *P-*value for interaction | N | β-CTX  β (95%CI) *P-*value | *P-*value for interaction |
| --- | --- | --- | --- | --- | --- | --- |
| Age |  |  | 0.369 |  |  | 0.307 |
| Low | 240 | 0.04 (-0.04, 0.12) 0.365 |  | 207 | -0.14 (-0.34, 0.05) 0.145 |  |
| Middle | 275 | 1.37 (-0.07, 2.80) 0.090 |  | 179 | -0.34 (-0.54, -0.14) 0.001 |  |
| High | 256 | 1.23 (-0.20, 2.66) 0.057 |  | 204 | -0.07 (-0.24, 0.09) 0.372 |  |
| BMI |  |  | 0.484 |  |  | 0.147 |
| Low | 258 | 0.03 (-0.05, 0.12) 0.437 |  | 188 | -0.15 (-0.31, -0.00) 0.049 |  |
| Middle | 278 | 0.09 (0.01, 0.16) 0.022 |  | 191 | -0.22 (-0.44, 0.00) 0.057 |  |
| High | 235 | 0.05 (-0.04, 0.13) 0.302 |  | 211 | -0.22 (-0.39, -0.05) 0.014 |  |
| Triglycerides |  |  | 0.540 |  |  | 0.935 |
| Low | 245 | 0.11 (0.03, 0.18) 0.006 |  | 158 | -0.22 (-0.41, -0.02) 0.030 |  |
| Middle | 264 | 0.06 (-0.02, 0.14) 0.118 |  | 198 | -0.22 (-0.39, -0.06) 0.009 |  |
| High | 262 | 0.04 (-0.05, 0.13) 0.375 |  | 234 | -0.12 (-0.29, 0.05) 0.175 |  |
| ALT |  |  | 0.912 |  |  | 0.886 |
| Low | 284 | 0.04 (-0.03, 0.12) 0.233 |  | 208 | -0.18 (-0.36, 0.01) 0.067 |  |
| Middle | 249 | 0.05 (-0.04, 0.14) 0.294 |  | 195 | -0.23 (-0.40, -0.06) 0.009 |  |
| High | 238 | 0.09 (0.01, 0.17) 0.026 |  | 187 | -0.11 (-0.29, 0.07) 0.228 |  |
| UA |  |  | 0.562 |  |  | 0.764 |
| Low | 264 | 0.06 (-0.03, 0.14) 0.223 |  | 210 | -0.27 (-0.45, -0.09) 0.005 |  |
| Middle | 262 | 0.06 (-0.01, 0.14) 0.093 |  | 213 | -0.13 (-0.27, 0.02) 0.092 |  |
| High | 245 | 0.10 (0.02, 0.18) 0.011 |  | 167 | -0.18 (-0.39, 0.02) 0.082 |  |
| Diabetes |  |  | 0.063 |  |  | 0.771 |
| Yes | 25 | 0.23 (0.04, 0.41) 0.033 |  | 24 | -0.46 (-1.39, 0.48) 0.357 |  |
| No | 746 | 0.06 (0.01, 0.11)0.016 |  | 566 | -0.20 (-0.31, -0.10) 0.000 |  |
| Hypertension |  |  | 0.439 |  |  | 0.458 |
| Yes | 103 | -0.01 (-0.17, 0.16) 0.942 |  | 75 | -0.22 (-0.53, 0.08) 0.153 |  |
| No | 668 | 0.07 (0.03, 0.12)0.003 |  | 515 | -0.18 (-0.29, -0.08) 0.001 |  |
| Monocyte |  |  | 0.147 |  |  | 0.347 |
| Low | 230 | -0.00 (-0.10, 0.09) 0.973 |  | 222 | -0.29 (-0.47, -0.12) 0.001 |  |
| Middle | 296 | 0.10 (0.02, 0.18) 0.013 |  | 237 | -0.02 (-0.18, 0.13) 0.774 |  |
| High | 243 | 0.09 (0.02, 0.16) 0.013 |  | 128 | -0.22 (-0.46, 0.02) 0.078 |  |
| Hemoglobin |  |  | 0.494 |  |  | 0.778 |
| Low | 269 | 0.08 (0.01, 0.15) 0.028 |  | 143 | -0.39 (-0.62, -0.16) 0.001 |  |
| Middle | 241 | 0.06 (-0.03, 0.14) 0.173 |  | 216 | -0.09 (-0.24, 0.06) 0.248 |  |
| High | 259 | 0.04 (-0.05, 0.12) 0.398 |  | 228 | -0.20 (-0.38, -0.02) 0.027 |  |
| Platelet |  |  | 0.682 |  |  | 0.211 |
| Low | 266 | 0.09 (0.02, 0.17) 0.019 |  | 200 | -0.18 (-0.36, -0.00) 0.047 |  |
| Middle | 253 | 0.04 (-0.04, 0.11) 0.339 |  | 203 | -0.18 (-0.33, -0.03) 0.020 |  |
| High | 250 | 0.06 (-0.02, 0.15) 0.155 |  | 184 | -0.23 (-0.45, -0.02) 0.032 |  |
| PTH |  |  | 0.262 |  |  | 0.676 |
| Low | 247 | 0.11 (0.01, 0.21) 0.032 |  | 218 | -0.19 (-0.31, -0.06) 0.006 |  |
| Middle | 270 | 0.05 (-0.02, 0.12) 0.158 |  | 203 | -0.13 (-0.31, 0.04) 0.136 |  |
| High | 254 | 0.06 (-0.12, 0.13) 0.095 |  | 169 | -0.33 (-0.61, -0.06) 0.020 |  |

Abbreviations: APOA1, apolipoprotein A1; Lumber BMD, lumbar spine bone density; β-CTX, C-terminal telopeptide of type I collagen; BMI, body mass index; ALT, alanine aminotransferase; UA, uric acid; PTH, parathyroid hormone; HDL, high-density lipoprotein; LDL, low-density lipoprotein; FAR, fibrinogen/albumin ratio.
